# Claudin 1 and Claudin 7 Gene Polymorphisms and Protein Derangement are Unrelated to the Growth Pattern and Tumor Volume of Colon Carcinoma

**Published:** 2010-06

**Authors:** Hahn-Strömberg Victoria, Edvardsson Henrik, Bodin Lennart, Franzén Lennart

**Affiliations:** 1 *Department of Clinical Medicine, Örebro University, Örebro and Dept. of Laboratory medicine, Örebro University Hospital, Örebro, Sweden;*; 2 *Department of Clinical Pathology, Karlstad, Sweden;*; 3 *Department of Statistics and Epidemiology, Örebro University Hospital, örebro, Sweden;*; 4 *Department of Statistics, Örebro University, Örebro, Sweden;*; 5 *Department of Pathology and Cytology, Aleris Medilab, Täby, Sweden*

**Keywords:** celladhesion, claudin, colon carcinoma, complexity index, polymorphism tight junction, tumor growth

## Abstract

Tight junctions together with adherens junctions are important for preserving tissue integrity. In tumors the normal tissue structure is lost which results in a disorganization and change of phenotype. In this study we assessed the complexity of the invasive front of colon carcinoma using an objective morphometrical technique based on the estimation of fractal dimension and number of free tumor cell clusters. The complexity of the invasive front was correlated to Claudin 1 and Claudin 7 protein expression as well as genetic polymorphisms of their genes. Thirty-three colon carcinomas were used. Images from the invasive front of the tumors were captured and used to calculate a complexity index of the invasive front. The tight junction proteins Claudin 1 and Claudin 7 were stained immunohistochemically in the tumor and in the surrounding normal mucosa. Screening of their genes was performed using DNA sequencing. A significant aberration of protein expression was seen for both Claudin 1 and Claudin 7 compared to normal mucosa. Both homozygous and heterozygous polymorphisms in exon 2 of claudin 1 were found. In claudin 7 a homozygous polymorphism was seen in exon 4. All individuals with tumors that showed either of these polymorphisms also showed the same polymorphism in the adjacent normal mucosa. A significant correlation was found between polymorphisms in CLDN 7 and tumor differentiation *p*<0.02. However no correlations were found to Complexity Index, tumor size, localization or tumor stage (pT and pN). The results show that there is a perturbed expression of claudin 1 and claudin 7 proteins in colon tumors compared to normal mucosa. A high incidence of polymorphisms was found in normal tissue and tumors. It remains to be shown if these polymorphisms are coupled to the occurrence of colon carcinomas.

## INTRODUCTION

The growth pattern of colorectal carcinoma is an important feature when assessing the prognosis. There are two different growth patterns of colorectal tumors, an expansive pattern where the invasion front is smooth and even and an infiltrative pattern showing an irregular front with tumor cell islands of various sizes. We have earlier defined a complexity index based on computerized morphometry that objectively gives the degree of complexity of the invasive front of the tumor (1).

All biological material follows fractal geometry through self similarity as do tumors. Fractal geometry has been used efficiently in different areas like molecular biology, bone, vascular and tumor pathology (2). For example, in pathology fractal dimension has shown to be able to differentiate between tubular, tubuvillous and villous adenomas of the colon as well as differentiate severe dysplasia and cancer from benign conditions in the epithelial connective tissue interface in the floor of the mouth (3, 4). By using fractal dimension and counting the number of free tumor cell islands we can quantitatively calculate the complexity index value of the tumor with a reproducible result.

Different adhesion proteins are involved in the invasion of colorectal cancer for ex, catenins, cadherins, matrix metalloproteinases and the tight junction proteins claudin and occludin (5, 6). They are transmembrane protein structures whose function is to build a barrier that controls the flow of molecules in the intercellular space (7, 8) and to take part in cell adhesion. Claudin 1 is normally expressed in different kinds of epithelial tissue and has been found to be upregulated in colorectal carcinoma (9) it has also been suggested as a target of the Beta-catenin/Tcf signaling (10, 11). Reduced claudin 1 expression has been associated with loss of differentiation in prostatic carcinoma and has been suggested to be involved in the transformation of biological behaviours in gastric carcinoma (12, 13). Upregulation of claudin 7 has been described as an early event in gastric tumorigenesis and is seen in dysplastic gastric glands (14).

Lioni *et al* suggests that claudin 7 regulates the expression of E-cadherin in esophageal squamous cell carcinoma, so that dysgregulation of claudin 7 leads to loss of E-cadherin expression and increased invasiveness (15). It has been suggested as a prognostic marker for colorectal carcinoma both independently or together with other markers like EpCAM and CD44 (16, 17).

We have earlier described a disturbed expression of the adhesion proteins Beta-catenin, E-cadherin, claudin 2 and occludin in colon carcinoma. This was unrelated both to polymorphisms in their genes and to the growth pattern and tumor volume of the tumors (18).

In this study we relate the expression of the tight junction proteins claudin 1 and claudin 7 and the nucleotide sequence of their genes to the complexity of the invasive border of colon carcinoma. Protein expression was assessed semiquantitatively using immunohistochemical staining. Parts of the invasive border were cut out by laser micro dissection (LMD) and analyzed for mutations in the corresponding genes by DNA sequencing and computerized morphometry was used to objectively determine the complexity of the invasive border.

## MATERIALS

Archived formalin fixed and paraffin embedded tissue from 33 samples from whole mount tissue section diagnosed with colorectal carcinomas were used. Two samples from each of the tumors including the invasive border were used for immunohistochemical staining, lasermicrodissection, PCR and computer image analysis. For tumor volume assessment the whole mount tissue section was used in order to achieve a correct value for the whole tumor. The samples were blinded. No mucinous carcinomas were included. The study was approved by the ethics committee at Örebro University Hospital Sweden.

## METHODS

### Immunohistochemistry

From each whole mount tissue section, two areas of the tumor was cut out and used for immunohistochemical staining. Immunohistochemistry was performed using the Envision technique (peroxidase) according to the manufacturer’s protocol. 4 micron sections of the tumors were mounted on polylysin coated microscopy slides for immunohistochemistry. The sections were deparaffinised in xylene twice for 10 min, dehydrated in a descending series of ethanol (99%, 96%, 70%) followed by washes in distilled water. Antigen retrieval was achieved by heating the samples in TE (Tris EDTA) buffer, pH9.0 ± 0.2 in a microwave oven at 650 W for 30 minutes. The sections were then washed in distilled water.

Staining was performed using a Dakos Techmate and DAB Envision according to the manufacturer’s protocol (Dako, Denmark). The slides were incubated with the antibodies for 30 minutes. The primary antibodies used were anti-claudin 1 (rabbit), Abcam, Cambridge, UK, dilution 1:200, anti-claudin 7 (5D10F3) Zymed, San Francisco, USA, dilution 1:1000 and anti-cytokeratin (Cam 5.2) BD Biosciences, San José, USA, dilution 1:25. Sections were transferred through ascending ethanol series and xylene before mounting and evaluated under a light microscopy.

### Evaluation of staining

Slides were consecutively numbered and anonymous to the observer. All slides were stained simultaneously in a DakoTechmate with a control slide that was exposed only to the secondary antibody. The immunoreactivity was assessed by the author (VH-S). The extent of staining of Claudin 1 and Claudin 7 was graded semiquantitatively into 4 categories where 0=0-10%, 1=10-50%, 2=50-80% and 3=80-100% stained cells. The distribution of the staining was localized to the membrane. This was performed both in sections from tumor and normal mucosa. The whole tissue was assessed in both sections in the center and in the invasive front of the tumor and an average percentage of the extent of staining score was made (19). Normal colon epithelium showed an even staining and there was very little discrepancy between the samples.

### Tumor volume assessment

**Figure 1:**
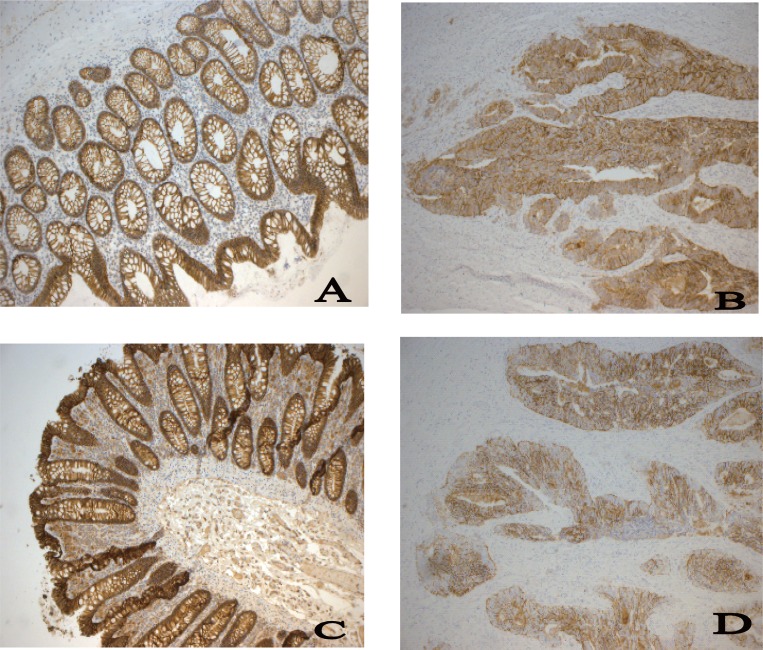
Photomicrographs of normal colon mucosa (a and c) and colon carcinoma (b and d) stained immunohistochemically for Claudin 1 (a and b) and Claudin 7 (c and d). An intense staining of the membrane and cytoplasm is seen in the normal mucosa while a weaker staining is seen in tumor cells.

Tumor slices with different thickness (4-11 mm) were processed for whole mount tissue sectioning and a thickness of 7 mm was chosen. This thickness preserved tissue integrity best and slices were easy to handle during dehydration and embedding in paraffin. A median of 7 slices were obtained from the 33 tumors (range 3-16). Whole mount sections were then made and stained with Hematoxyline-eosin. The outline of the tumor was marked with ink (Figure 1) and the whole mount sections scanned in a flat bed scanner. The images were fed into a computer and the cross sectional area of the tumor was then measured in each whole mount section using the image analysis software Adobe Photoshop CS2. Knowing the distance between sections and the area of each section the volume of each tumor was calculated using Cavalieri’s principle (20).

### Laser micro dissection

For laser micro dissection 10-micron sections were mounted on slides for membrane-based laser micro dissection (Leica Microsystems GmbH, Wetzlar, Germany). The slides were then manually stained with anti-cytokeratin CAM 5.2 as described above. A 1mm^2^ piece from the invasive front of the tumor samples was extracted to ensure that only tumor cells from the invasive front were processed. If the sample contained a small amount of tumor cells, several LMD pieces were pooled together in the same tube. A similar procedure was used to obtain normal mucosa for analysis.

### PCR

The whole gene consisting of 4 exons in both Claudin 1 and Claudin 7 was analyzed. The size of the amplicons ranged from 100-112 bp. Primers were designed using the Primer 3 programme (http://www.embnet.sk/cgi-bin/primer3_www.cgi). If possible parts of the intron section was used in the primers. DNA was extracted using proteinase K according to manufacturers protocol (Qiagen) and the amplification was performed in an optimized PCR according to the following protocol á 50 cycles on a thermocycler (Eppendorf gradient) at 95°C denaturation for 1 min, 56-60°C annealing, depending on the amplicons for 1 min and 72°C extension for 1 min. PCR was performed using a volume of 50 μL containing 1-10 ng of genomic DNA in a buffer containing 1.5-2.0 mM MgCl_2_, 200 uM each of deoxyribonucleoside triphosphate, 5pmol of each primer and 0.5 units of Taq Gold polymerase (Applied Biosystems, Foster City, USA). Primer sequences and Tm temperatures can be seen in Table 1.

**Table 1:** Primer sequences used for PCR and DNA sequencing reactions

Primer sequence 5´-3´ CLDN1	Name of amplicon	Size (bp)	Annealing temp (°C)

F:tctccgccttctgcacct	CLDN1ex1amp1	101	60
R:aggaaggcgagaatgaagc			
F:cttcctgggatggatcgg	CLDN1ex1amp2	112	59
R:ccacagcccctcgtacat			
F:catgtacgaggggctgtg	CLDN1ex1amp3	100	59
R:ggtgcactcactgctcagat			
F:tttctgccaggcacattg	CLDN1ex2amp1	104	56
R:ttcatacacttcatgccaacg			
F:cgttggcatgaagtgtatgaa	CLDN1ex2amp2	111	56
R:ggggcacagcctctattacc			
F:ttttgaatttctataggtctggcta	CLDN1ex3	107	59
R:cgttacctggcattgactg			
F:gcacatgggtttttcctttt	CLDN1ex4amp1	101	54
R:acagcaaagtagggcacctc			
F:tgccctactttgctgttcc	CLDN1ex4amp2	105	59
R:ctgtgtcacacgtagtctttcc			

### DNA Sequencing

For DNA sequencing the PCR product was purified using Ethanol/EDTA/Sodium Acetate Precipitation and sequencing was performed using ABI Prism Big Dye Terminator cycle sequencing Ready Reaction Kit v1.1 on the ABI 3100 and ABI 3130xl (Applied Biosystems, Foster City, USA). The DNA sequences were subjected to NCBI Blast (National Centre for Biotechnology Information; http://www.ncbi.nlm.nih.gov) for verification of the amplified amplicons.

### Computer Image Analysis

Images were captured from the tumor-stromal interface in specimens stained immunohistochemically for CAM 5.2 and fed into a computer (objective 10X). Later image processing including thresholding of the images was performed so that (i) all immunohistochemically stained areas became black and (ii) the tumor border was outlined with a black line. The images with black tumor cells were used for counting the number of Tumor Cell Clusters and the images with the tumor outlined were used for the estimation of the Fractal Dimension. These two characteristics were then used in a “tree analysis” rendering the tumors a number between 1 and 5 indicating the degree of complexity of the invasive margin (1 indicates a smooth border and 5 a highly irregular border). As a mean, 9.3 (range 5-16) images were analyzed per tumor and the mean values of Fractal Dimension and number of Tumor Cell Clusters of each tumor was used for estimation of the Complexity Index.

### Statistical methods

Analysis of variance (ANOVA) was used to analyze differences in volume between groups of objects specified by the different categorizations for tumor complexity, CLDN1T and CLDN7T. For some of these analyzes we had to recode the variables into fewer categories to avoid cells with very few observations. In those cases where correlations were evaluated we used Spearman’s rho due to weaker distributional properties of the analyzed variables. To analyze differences in the distributions of CLDN1Tumor vs CLDN1Normal mucosa and CLDN7Tumor vs. CLDN7Normal mucosa, we used the chi-square test for homogeneity in distribution. The calculation was performed with permutation tests that were especially adopted to handle small samples since the number of cells was too small for the asymptotic chi-square test. *P*-values <0.05 were classified as statistical significance. All statistical testing was done with SPSS (version 15, SPSS Inc, IL) or StatXact (Version 8, Cytel Inc, MA).

## RESULTS

Altogether 33 tumors were examined. Of these seven were located in the caecum, eight in the ascending colon, one in the right flexure, four in the transversum, one in the left flexure and twelve in the sigmoid colon. Two tumors were well differentiated, twenty-one moderately differentiated and eleven were poorly differentiated. The degree of differentitation was made according to WHO’s classification (21) Using the TNM system one tumor was staged pT1, two pT2, twenty pT3 and ten pT4. Fifteen tumors had no lymph node metastases (pN0), ten were staged pN1 and eight pN2. The average tumor volume was 24.1 cm^3^ (range 1.54-94.4 cm^3^). The complexity index was assessed after morphometrical processing of images from the tumor invasive front in specimens stained immunohistochemically for cytokeratin. Of the 33 tumors analyzed, 26 had a Complexity Index of 1, six had a Complexity Index of 2 and one sample had a Complexity Index of 5.

Since some tumors show different growth patterns depending on which area is assessed we cut out two areas and calculated the complexity index of these areas in order to see if there was a difference in tumor complexity between calculating the complexity index of the whole mount tumor section as well as two areas of the tumor.

Immunohistochemical staining was performed for claudin 1 and claudin 7. A strong and even membrane staining was seen in the normal mucosa while the tumors showed a varying distribution of the proteins. This variation was mostly seen in tumors that were poorly differentiated which showed a more fragmented and weaker membranous pattern. The results of the semi quantitative analysis of the staining are shown in Table 2. The aberrations were significant for both claudin 1 and claudin 7 (*p*<0.0001). There was no correlation to tumor differentiation, volume, localization, Complexity Index, or stage (pT and pN) for protein expression of claudin 1 or claudin 7.

**Table 2:** Results of immunohistochemical staining Claudin 1 and Claudin 7 in normal colon epithelial cells and colon carcinoma tumor cells

	Immunoreactivity score			
	0	1	2	3

Claudin 1 normal mucosa				33
Claudin 1 tumor		1	15	17
Claudin 7 normal mucosa				33
Claudin 7 tumor		3	12	18

**Figure 2:**
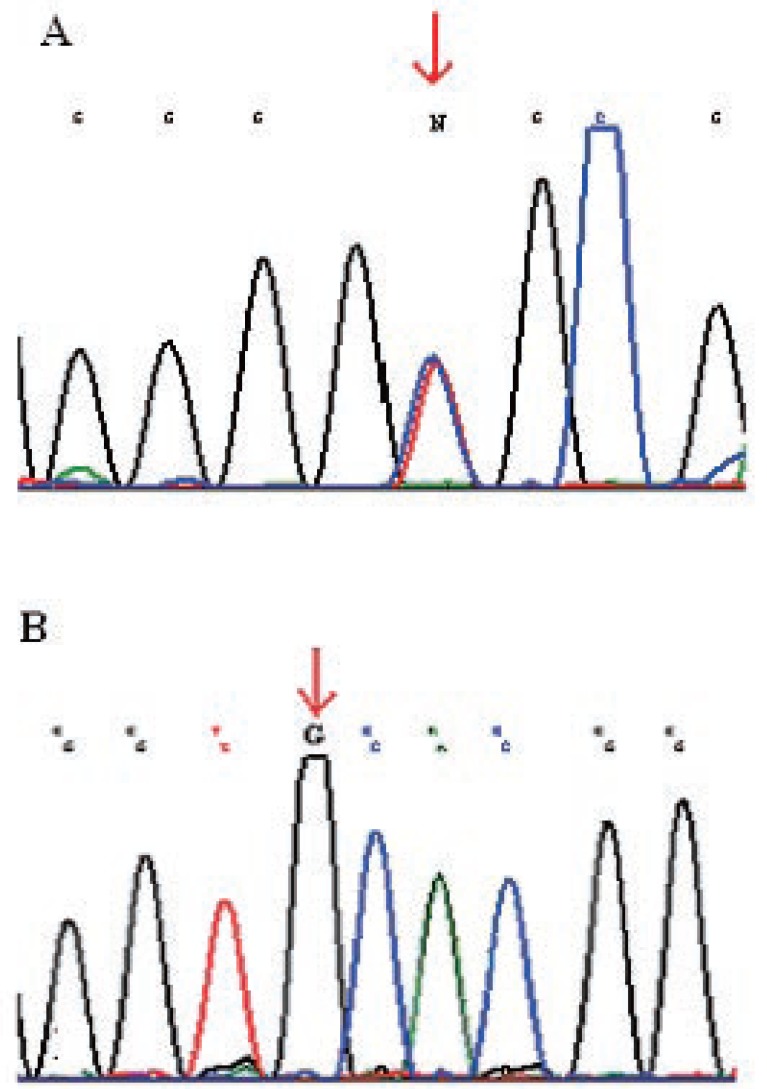
Chromatogram of DNA sequencing of the CLDN1 gene (a) showing a heterozygous SNP in exon 2 and CLDN7 (b) showing a homozygous SNP in exon 4.

DNA sequencing was performed on 26 tumors for CLDN1 and CLDN7. Twenty of these had a Complexity Index of 1, five a Complexity Index of 2 and one a Complexity Index of 5. In claudin 1, 17 out of 26 samples showed a homozygous A/G (Gly123Gly) polymorphism in exon 2 rs9869263 and eight showed a heterozygous AG polymorphism. The distribution of the polymorphisms among the different growth patterns is shown in Table 3. The polymorphisms were always seen in both tumor and normal mucosa of the same individuals. Only one of the 26 individuals showed no polymorphisms in claudin 1 exon 2, either homozygous or heterozygous. No correlations were seen for polymorphisms in claudin 1 and differentiation, tumor localization, tumor volume, pT and pN (Figure 2).

**Table 3:** Results of DNA sequencing of the genes of Claudins 1 and 7

	Complexity Index				
	1	2	3	4	5

Claudin 1 homozygous polymorphism	13	3	-	-	1
Claudin 1 heterozygous polymorphism	6	2	-	-	-
Claudin 7 homozygous polymorphism	12	2	-	-	1

The distribution of tumors with different complexity indices and polymorphisms are shown.

In claudin 7, fifteen tumors and normal mucosa from the same individuals showed a homozygous polymorphism A/G (Val197Ala) rs4562 in exon 4. The remaining eleven showed no polymorphisms in this gene either in either the tumor or in the normal mucosa.

The distribution of the polymorphisms among the different growth patterns is shown in Table 3. There was a significant correlation between polymorphisms in CLDN 7 and tumor differentiation *p*<0.02. However no correlation was found between claudin 7 gene polymorphisms and Complexity Index, tumor size, localization, pT or pN.

## DISCUSSION

In this study we have assessed the growth complexity of 33 colon carcinomas by estimating the Fractal Dimension of the invasive front and the number of tumor Cell Clusters using computerized morphometry. A complexity index was created and correlated to the expression of the tight junction proteins claudin 1 and claudin 7 after immunohistochemical staining. A mutation analysis of their corresponding genes was performed using DNA sequencing. Only carcinomas from the colon were used since most patients with rectal carcinomas obtain local radiation to the tumor preoperatively.

The Claudin family include about 24 member proteins. Claudins span the cellular membrane 4 times with the N-terminal and the C-terminal end both located in the cytoplasm (8).Their main function is to prevent leakage and diffusion of molecules across epithelial barriers but they also contribute to the adherence of cells. The localization and expression of Claudins may differ depending on the type of tissue and neoplasm. In malignant tumors, tight junctions frequently show structural and functional abnormalities (22, 23). For example increased expression of claudins 3 and 4 have been seen in ovarian and prostate cancer (24, 25) while claudin 1 expression has been found to be upregulated and of prognostic significance in colon cancer (11, 26). Claudin 1 as well as other claudins have shown in other studies a varied expression from low to elevated using immunohistochemistry in colon carcinoma depending on degree of differentiation (26). However there are only a few studies made on the protein expression of claudins in colon carcinoma and more are needed to fully understand the involvement of claudins. Also, the evaluation of the immunohistochemistry staining varies in the studies, sometimes the intensity of the staining is evaluated and sometimes the extent and/or localisation or both are evaluated.

According to Soini claudin 1, 2, 3, 4, 5 and 7 can be used as epithelial differentiation markers and can be used to distinguish epithelial tumors from each other (17). Claudins have also been suggested as possible biomarkers and targets for cancer therapy (27-29). Gröne *et al* (9) found that claudin 1 is frequently overexpressed in colorectal carcinoma compared to normal tissue by using RT-PCR but there was no correlation to clinicopathological parameters.

We have earlier shown disturbed protein expression of Beta-catenin, E-cadherin, occludin and claudin 2 compared to normal surrounding mucosa in colon carcinoma. However we did not find any polymorphisms in those genes that could account for the aberrant protein pattern (18). A correlation between colon carcinoma volume and growth pattern was found but no relation to the disturbed adhesion protein expressions(18). An aberrant expression of tight junction proteins claudin 1 and claudin 7 in colon carcinoma compared to normal mucosa was found in this study. Furthermore, both homozygous and heterozygous polymorphisms were found in these genes in both the tumor and the adjacent normal mucosa of the same individuals. No individual had mutations in either the tumor or the normal mucosa alone. However, there was no correlation between the occurrence of aberrant Claudin protein expression and the polymorphisms. A significant correlation was found between polymorphisms in CLDN 7 and tumor differentiation *p*<0.02. No other correlations were found to tumor differentiation, volume, localization, Complexity Index, or stage (pT and pN).

In conclusion, no correlations were found between the aberrant protein expression or gene derangements and tumor complexity. Since evaluation of immunohistochemical staining is semiquantitative a morphometric method could be used in order to achieve a quantitative result or by using Western Blot. An interesting finding was a high frequency of polymorphisms in the genes of the Claudins, both in normal tissue and tumors. If this frequency is low in the general population, the results could indicate an increased risk of colon carcinoma in individuals with these polymorphisms. Further studies of tight junction proteins genes with larger population samples are needed to better understand if these mutations are of importance in colorectal carcinogenesis.
